# Distribution of *Candida albicans* and non-*albicans Candida* species isolated in different clinical samples and their in vitro antifungal suscetibity profile in Ethiopia

**DOI:** 10.1186/s12879-020-4883-5

**Published:** 2020-03-19

**Authors:** Elias Seyoum, Adane Bitew, Amete Mihret

**Affiliations:** 1 Ethiopian Public Health Institute, Clinical Bacteriology and Mycology Research Case Team, Addis Ababa, Ethiopia; 2 Department of Medical Laboratory Sciences, College of Health Sciences, Addis Ababa University, Addis Ababa, Ethiopia

**Keywords:** *Candida albicans*, Non-*albicans Candida* species, Antifungal drugs, drug susceptibility profile of *Candida* species

## Abstract

**Background:**

The spectrum of yeasts and their antifungal susceptibility profile are poorly known and treatment of fungal disease has remained empirical. The aim of this study is to determine the spectrum and antifungal susceptibility profile of yeasts particularly of *Candida* species.

**Methods:**

A descriptive study on the composition of *Candida* species and antifungal susceptibility profile were conducted from January 2018 to September 2018. Clinical samples collected from different sites were cultured on Sabouraud dextrose agar and incubated for an appropriate time. Identification of yeast isolates and their antifungal susceptibility profile were determined by the VITEK 2 compact system. Descriptive statistics such as frequency and percentage of *Candida* species were calculated using SPSS version 20.

**Results:**

Of 209 yeasts recovered, 104(49.8%), 90 (43.1%), 15(7.2%) were *C. albicans,* non *albicans Candida* species, and other yeasts, respectively. Among non *albicans Candida* species, *Candida krusei* was the commonest isolate. Of other yeast groups, 66.7% was represented *by Cryptococcus laurentii.* Regardless of *Candida* species identified, 85.6, 3.9, and 10.5% of the isolates were susceptible, intermediate, and resistant to fluconazole, respectively. *C krusei* was 100% resistant to the drug. Voriconazole demonstrated the greatest antifungal activity against *Candida* isolates in which 99.4% of *Candida* isolates were susceptible. The susceptibility and the resistance rate of *Candida* isolate to both caspofungin and micafungin were the same being 96 and 4% respectively. However, micafungin was more potent than caspofungin. The susceptibility, resistant, and intermediate rates of yeasts against flucytosine were, 86.2, 6.6, and 7.2%, respectively.

**Conclusions:**

The present study demonstrated the distribution of *Candida* species in different clinical specimens where the isolation rate of non-*albicans Candida* species was comparable to *Candida albicans*. The high resistance rate of *C. krusei* to fluconazole and flucytosine may demonstrate that the treatment of candidiasis empirically is questionable.

## Background

*Candida* species are the most common cause of fungal infections, leading to a range of life-threatening invasive diseases such as blood stream candidiasis to non-life-threatening mucocutaneous candidiasis such as genitourinary candidiasis, vulvovaginal candidiasis, and oropharyngeal candidiasis [1]. They are also an important cause of superficial mycosis such as onychomycosis. Among fungal infections, invasive candidiasis is commonly associated with high morbidity and mortality rate. For example, *Candida* species are among the top ten pathogens causing bloodstream infections [1], resulting in increased death rate, patient hospitalization, and healthcare costs [2]. Mucocutaneous candidiasis is one of the indirect signs for cell-mediated immunodeficiency and estimated to have more than 90% positive predictive value for invasive candidiasis [3].

Until recently, *C. albicans* was recognized as the commonest species causing most of the cases of candidiasis. However, in the last few decades, several studies reported that there has been a progressive shift from a predominance of *C. albicans* to non-*albicans Candida* species (NAC) such as *C. tropicalis*, *C. glabrata* and *C. krusei* [4–6]. NAC species have been reported to be a major cause of fungal opportunistic infection [7, 8]. An increase in opportunistic fungal infections is the result of an increase in the number of immune-compromised patients. Excessive use of broad-spectrum antibiotics, metabolic disorders, and the emergence of AIDS are among the various contributing factors for an increase in opportunistic fungal infections [9–12].

Development of resistance to azoles, the treatment of choice for fungal infections, mainly by NAC species, differences in drug susceptibility profile among yeast isolates, and frequent isolation of emerging yeasts (i.e., NAC species) in clinical samples initiated the use of accurate species identification and in vitro susceptibility testing methods [13]. It has been recognized that many phenotypic automated and molecular yeast identification techniques as well as standardized methods for antifungal susceptibility testing are available in many laboratories. Unfortunately, in Ethiopia, even the simplest yeast diagnostic methods such as chromogenic medium and carbohydrate assimilation as well as simple antifungal susceptibility testing procedures are lacking. Consequently, the spectrum of yeasts and their antifungal susceptibility profile are poorly known and treatment of fungal disease has remained empirical. Against this back ground, the aim of this study is to determine the spectrum and antifungal susceptibility profile of yeasts particularly of *Candida* species recovered from different clinical samples from patients referred to Arsho Advanced Medical Laboratory for routine tests. Identification and sensitivity testing were performed by using the VITEK 2 compact system. The VITEK 2 compact system (bioMérieux) is a fully automated system that is accurate in the identification of yeasts and evaluating their drug susceptibility profile simultaneously [14, 15].

## Methods

### Study population and period

A study on the composition of yeast species and antifungal susceptibility profile of yeasts recovered in culture from different clinical samples was carried out from January 2018 to September 2018 at Addis Ababa, Ethiopia.

### Sample collection

A total of 776 clinical samples including throat swab (119), nail scrapings (154), sputum (142), corneal scrapings (146), and vaginal swabs (215) were collected from patients referred to Arsho advanced medical laboratory according to standard procedures related to each sample. One clinical sample per patient was collected. All clinical samples were inoculated on to Sabouraud dextrose agar (Oxoid, Basingstoke, UK) to which 50 μg/1 ml gentamicin is incorporated. Inoculated plates were incubated at a temperature of 37^0^ C for at least 72 h aerobically. Then yeast isolates were transferred to Brain heart infusion broth (Oxoid, Basingstoke, UK) containing 10% glycerol and transferred to the Department of Medical Laboratory Technology, College of health Sciences, Addis Ababa University and stored at minus 80^o^ deep freezer until use.

### Identification and antifungal susceptibility testing

The viability of yeast cultures that have been stored at minus 80^o^ deep freezer was checked by inoculating them on to Sabouraud dextrose agar (Oxoid, Basingstoke, UK). Identification of yeast cultures and their in vitro antifungal susceptibility profile were determined by the automated VITEK 2 compact system (bioMérieux, France) using YST-21343 and AST-YS07 cards following the instruction of the manufacturer at the Ethiopian Public Health Institute situated in the same campus with the Department of Medical Laboratory Science. Briefly, inoculum suspensions for the VITEK 2 were prepared in sterile saline at turbidity equal to 2.0 McFarland standards, as measured using a DensiChek instrument (bioMérieux). The YST-21343 and AST-YS07 cards were automatically filled with the prepared culture suspension, sealed, and incubated by the VITEK 2 instrument. The cards were incubated at 35.5 °C for 18 h, and data were collected at 15-min intervals during the entire incubation period and final identification and break point minimum inhibitory concentration (MIC) values used to categories *Candida* species, susceptible, intermediate or resistant to each drug were investigated using version 7.0 software, an advanced expert system (AES) designed to evaluate the results produced by the VITEK 2 system. Identification of each isolate down to a species level assigned by the VITEK 2 compact system as excellent, very good, good, acceptable, or low- discrimination was taken as correct identification.

## Quality assurance

Clinical samples were inoculated after the performance of culture media and their sterility was checked following standard procedure. A standard strain of *C. albicans* (ATCC 10231) was used to evaluate the performance of the VITEK machine.

### Data analysis and interpretation

All data from the investigation were coded and analyzed using SPSS version 20. Descriptive statistics such as frequency and percentage of *Candida* species were calculated.

## Ethical clearance

All ethical deliberations and responsibilities were appropriately addressed, and the study was conducted after the approval of the Internal Review Board (IRB) of the Department of Medical Laboratory Sciences (DRERC/323/18/MLS) and after permission letter was obtained from Ethiopian Public Health Institute and Arsho Advanced Medical Laboratory private limited company. Informed written consent was obtained from the study participant ahead of data collection. Each participant was given the right to refuse or to participate in the study and even to withdraw him/her from the study at any time during the course of the study. All information recorded from the study subjects were kept confidentially. An assent form was complted and signed by a family member and/or adult guardian for patients under the age of 16 years.

## Results

A total of 209 yeasts were isolated from 776 different clinical samples (Table 1). Among the isolates, 84(40.2%) were recovered from vaginal swab while 52(24.9) were isolated from the oropharyngeal swab. The remaining 42(20.1%), 19(9.1%) and 12(5.7%) were isolated from sputum, corneal, and nail scrapings, respectively**.** The distribution of *Candida* with respect to clinical sample was variable where *C. albicans*, *C. famata,* and *C. kefyr* recovered in all clinical specimens. Of 209 yeasts recovered, 104(49.8%) were *C. albicans*, 90 (43.1%) were NAC species, and 15 (7.2%) were yeasts other than *Candida* species. Among NAC species, *C. krusei* 15.6%, *C. famata* 14.4%, *C. rugosa* 11.1%, and *C. lusitaniae* 10.0% were the commonest isolates. Out of yeasts other than *Candida* species *Cryptococcus laurentii* represented 66.7% *of* this group of yeasts.

**Table 1:** Distribution of yeast isolates per clinical samples

Clinical samples						
Species	Vaginal swab	Oropharyngeal	Nail	Eye Discharge	Sputum	Total (n, %)
*Candida albicans*	52	30	4	5	13	104
***Sub-total***						**104(49.8)**
***Non-albicans Candida***						
*C. krusei*	11	0	0	2	1	14(15.6)
*C. famata*	2	4	5	1	1	13(14.4)
*C. rugosa*	0	2	1	1	6	10(11.1)
*C. lusitaniae*	3	2	3	1	0	9(10)
*C. dubliniensis*	5	0	0	0	2	7(7.8)
*C.lipolytica*	2	4	0	0	0	6(6.7)
*C. parapsilosis*	2	4	0	0	0	6(6.7)
*C. ciferrii*	2	0	0	0	4	6(6.7)
*C. kefyr*	1	2	1	1	1	6(6.7)
*C. guilliermondii*	0	3	1	1	0	5(5.6)
*C. pelliculosa*	1	0	3	0	0	4(4.4)
*C. glabrata*	2	0	0	0	0	2(2.2)
*C. intermedia*	0	0	1	0	0	1(1.1)
*C. utilis*	0	1	0	0	0	1(1.1)
***Sub total***		**.**				**90(43.1)**
**Other yeasts**						
*Cryptoccocus laurenti*	0	0		0	10	10(66.7)
*C. neoformans*		0		0	4	4(26.7)
*Trichosporn mucoides*	1	0	0	0	0	1(6.7)
***Sub-total***						**15(7.2)**
**Grand Total (n, %)**	**84(40.2)**	**52(24.9)**	**19(9.1)**	**12(5.7)**	**42(20.1)**	**209**

The proportion of *C. albicans* to NAC species and the first three predominant *Candida* species in this study in comparison with earlier studies between and within countries are presented in Table 2. The three predominant *Candida* species in our study in their descending order were *C. albicans, C. krusei, and C. famata.*

**Table 2:** The proportion of *C. albicans* to NAC species and the first 3 dominant Candida species isolated in the current study compared to earlier studies between and with countries

			Clinical sample	Country		Reference
No, isolates	***C. albicans,*** n (%)	NAC n (%)			Dominate species	
194*	104(49.8%)	90 (43.1%)	**Various**	**Ethiopia**	***C. albicans, C. krusei, C. fabata***	*
**177**	139(78.5)	38(21.5)	**Oral (HIV patients**	**Ethiopia**	***C. albicans, C. glabrata,****C. tropicalis*	**Mulu et al, 2013** [16]
**81**	**51(**58.6)	**30(**41.4)	**Vaginal swab**	**Ethiopia**	***C. albicans, C. krusei,****C. dubliniesis*	**Bitew and Abebaw, 2018** [17]
**63**	**38(60.3)**	**25(39.7)**	**Vaginal swab**	**Egypt**	*C. albicans, C. glabrata, C. krusei*	ElFeky et al, 2016 [18]
**111**	**44(39.6)**	**67(60.4)**	**Various**	**India**	***C. albicans, C. krusei, C tropicalis***	**Mohandas & Balla 2011** [19]
**90**	**33(36.7)**	**57(63.3)**	**Various**	**India**	***C. albicans, C. tropicalis,****C. parapsilosis*	**Das et al 2016** [20]
**102**	**37(36.3)**	**65(63.7)**	**Various**	**India**	***C. tropicalis, C. albicans.****C. guilliermondii*	**Sida et al, 2017** [21]
**250**	**154(61.6)**	**96(38.4)**	**Oral cavity**	**Thailand**	*C. albicans, C. glabrata, C. tropicalis*	Muadcheingka&Tantivitayakul,2015 [22]
**108**	**61(56.5)**	**47(43.5)**	**Blood**	**Taiwan**	*C. albicans, C. glabrata, C. tropicalis*	**Chi et al,2011** [23]
**90**	**33(36.7)**	**57(63.3)**	**Various**	**India**	*C. tropicalis, C.albicans. C. glabrata*	Kaur et al, 2016 [24]
**103**	**80(77.8)**	**23(22.3)**	**Oral (HIV patients)**	**Brazil**	*C.albicans, C. tropicalis, C. parapsilosis*	Ribeiro, et al, 2015 [25]
**1062**	**573(54)**	**489 (46)**	**Various**	**Germany &Austria**	*C. albicans, C. glabrata, C. parapsilosis*	Schmalreck et al, 2012 [26]
**580**	**420(72.4)**	**160(27.6)**	**Vaginal swab**	**America**	*C. albicans, C. glabrata, C. parapsilosis*	Richter et al, 2005 [27]
**428**	**273(63.8)**	**155(36.2)**	**Various**	Iran	*C. albicans. C. tropicalis, C. parapsilosis*	Badiee et al, 2011 [28]
**103**	**43(42)**	**60(58)**	**Blood**	Latin America	*C. albicans. C. tropicalis, C. parapsilosis*	**Godoy et al, 2003** [29]

* = current study

### The antifungal profile of *Candida* isolates

The in vitro antifungal susceptibility profile of *Candida* species to the five antifungal drugs expressed as MICs in μg/ml is shown in Table 3. Among 194 *C. albicans* and NAC isolates, the MIC values of *C. famata* to all drugs tested and *C. ciferrii* to echinocandins were not determined by the VITEK. Regardless of *Candida* species identified, 85.6% were susceptible while 10% were resistant to fluconazole. The remaining 3.9% were intermediate to the agent. Hundred percent of *C. krusie*, 20% *C. rugosa,* 25% of *C. pelliculosa,* 17% *C. lipolytica* and 17% *C. ciferrii* demonstrated resistant to the agent. Furthermore, 2% *C. albicans, 28.6% C. dubliniensis, 10*% of *C. rugosa*, 17% *C.lipolytica*, and 17% *C. ciferrii were* intermediate to fluconazole. Voriconazole showed the greatest antifungal activity against *Candida* isolates in which all *Candida* isolates (99.4%) exhibiting susceptibility while one (0.6%) isolate of *C. ciferrii was* found out to be resistant. The susceptibility and the resistance rate of *Candida* isolates to both caspofungin and micafungin were the same being 96%). Four isolates of *C. albicans*, two isolates of *C. rugosa* and one isolate of *C. lipolytica* demonstrated resistance to the drugs. Similarly, 86.2% of yeast isolates were susceptible to flucytosine, 6.6% were resistant while 7.2% were intermediate to it. One isolate of *C. albicans* and 11 isolates of *C. krusei* were resistant to the drug.

**Table 3:** Over all percentage antifungal drug profile of *Candida* species

Species	Fluconazole			Voriconazole			Caspofungin			Micafungin			FLu cytosine		
N = 194	S	R	I	S	R	I	S	R	I	S	R	I	S	R	I
*C. albicans (104)*	98 (102)	0	2(2)	100(104)	0	0	96.2(100)	3.8(4)	0	96.2(100)	3.8(4)	0	96(100)	1(1)	3(3)
*C. krusie (14)*	0	14	0	100 (14)	0	0	100(14)	0	0	100(14)	0	0	21.4 (3)	78.6 (11)	0
*C. famata (13)*	*	*	*	*	*	*	*	*	*	*	*	*	*	*	*
*C. rugosa (10)*	70 (7)	20(2)	10(1)	100(10)	0	0	80(8)	20(2)	0	80(8)	20(2)	0	50 (5)	0	50(5)
*C. lusitaniae(9)*	100(9)	0	0	100(9)	0	0	100 (9)	0	0	100(9)	0	0	100(9)	0	0
*C. dubliniensis(7)*	71.4(5)	0	28.6(2)	100(7)	0	0	100(7)	0	0	100(7)	0	0	71.4(5)	0	28.6(2)
*C.lipolytica(6)*	66(4)	17(1)	17(1)	100(6)	0	0	83.3(5)	16.7(1)	0	83.3(5)	16.7(1)	0	66.7 (4)	0	33.3(2)
*C. parapsilosis (6)*	100(6)	0	0	100(6)	0	0	100(6)	0	0	100(6)	0	0	100(6)	0	0
*C. ciferrii (6)*	66(4)	17(1)	17(1)	83(5)	17(1)	0	*	*	*	*	*	*	83.3(5)	0	16.7(1)
*C. kefyr (6)*	100 (6)	0	0	100(6)	0	0	100(6)	0	0	100(6)	0	0	100(6)	0	0
*C. guilliermondii(5)*	100(5)	0	0	100(5)	0	0	100(5)	0	0	100(5)	0	0	100(5)	0	0
*C. pelliculosa(4)*	75(3)	25(1)	0	100(4)	0	0	100(4)	0	0	100(4)	0	0	100(4)	0	0
*C. glabrata (2)*	100(2)	0	0	100(2)	0	0	100(2)	0	0	100(2)	0	0	100(2)	0	0
*C. intermedia(1)*	100(1)	0	0	100(1)	0	0	100(1)	0	0	100(1)	0	0	100(1)	0	0
*C. utilis(1)*	100(1)	0	0	100(1)	0	0	100(1)	0	0	100(1)	0	0	100(1)	0	0
*Total yeast tested*	155	19	7	180	1	0	168	7	0	168	7	0	156	12	13
*%*	85.6	10.5	3.9	99.4	0.6	0	96	4	0	96	4	0	86.2	6.6	7.2

* = MIC values were not provided by the machine

Break points of different antifungal drugs against *Candida* Isolates are shown in Table 4. The MIC values of fluconazole, voriconazole, caspofungin, micafungin and, flucytosine against *C. albicans* were variable. Relatively, higher fluconazole MICs were found in NAC species than *C. albicans.* All Isolates of *C. krusei* were resistant to fluconazole at a concentration of ≥16 μg/ml. The potency of micafungin was better than that of caspofungin. Most isolates were susceptible to micafungin at the lowest dilution drug tested (0.06 μg/ml).

**Table 4:** Break points of different antifungal drugs against *Candida* Isolates

Species	Antifungal drug	Break points μg/ml		
		Susceptible	Intermediate	Resistant
	Fluconazole	1–4	16–32	0
	Voriconazole	0.12–0.5	–	–
*C. albicans*	Caspofungin	0.12–1	–	4
	Micafungin	0.06–0.5	–	4
	Flucytosine	1	8	64
	Fluconazole	0	–	16
	Voriconazole	0.12–0.25	–	–
*C. krursei*	Caspofungin	0.12–0.5	–	–
	Micafungin	0.06–0.25	–	–
	Flucytosine	1	16	64
	Fluconazole	*	*	*
	Voriconazole	*	*	*
*C. famata*	Caspofungin	*	*	*
	Micafungin	*	*	*
	Flucytosine	*	*	*
	Fluconazole	1–8	32	64
	Voriconazole	0.12	–	–
*C. rugosa*	Caspofungin	0.25	–	4
	Micafungin	0.06–0.12		4
	Flucytosine	1	16	–
	Fluconazole	1–2		
	Voriconazole	0.12–0.5	–	–
*C. lusitaniae*	Caspofungin	0.25–1	–	–
	Micafungin	0.06–1	–	–
	Flucytosine	1	–	–
	Fluconazole	1–8	32	–
	Voriconazole	0.12	–	–
*C. dubliniensis*	Caspofungin	0.12–0.25	–	–
	Micafungin	0.06–0.25	–	–
	Flucytosine	4	8	–
	Fluconazole	1–8	16	64
	Voriconazole	0.12	–	–
*C.lipolytica*	Caspofungin	0.12–0.25	–	≥ 4
	Micafungin	0.06–0.25	–	≥ 4
	Flucytosine	8	16	–
	Fluconazole	1	–	–
	Voriconazole	0.12	–	–
*C. parapsilosis*	Caspofungin	0.12–1	–	–
	Micafungin	0.25–2	–	–
	Flucytosine	1	–	–
	Fluconazole	1–8	16	64
	Voriconazole	0.12	–	32
*C. ciferrii*	Caspofungin	*	*	*
	Micafungin	*	*	*
	Flucytosine	2	8	–
	Fluconazole	1	–	–
	Voriconazole	0.12	–	–
*C. kefyr*	Caspofungin	.025–0.5	–	–
	Micafungin	0.06.0.12	–	–
	Flucytosine	1	–	–
	Fluconazole	1–4	16	–
	Voriconazole	0.12	–	–
*C. guilliermondii*	Caspofungin	0.25–1	–	–
	Micafungin	0.06–0.5	–	–
	Flucytosine	1	–	–
	Fluconazole	2	–	64
	Voriconazole	0.12–0.5	–	–
*C. pelliculosa*	Caspofungin	0.25	–	–
	Micafungin	0.06–0.12	–	–
	Flucytosine	1–4	–	–
	Fluconazole	4	–	–
	Voriconazole	0.12	–	–
*C. glabrata*	Caspofungin	0.25	–	–
	Micafungin	0.06	–	–
	Flucytosine	1	–	–
	Fluconazole	1	–	–
	Voriconazole	0.12	–	–
*C. intermedia*	Caspofungin	0.25	–	–
	Micafungin	0.12	–	–
	Flucytosine	1	–	–
	Fluconazole	2	–	–
	Voriconazole	0.12	–	–
*C. utilis*	Caspofungin	0.25	–	–
	Micafungin	0.12	–	–
	Flucytosine	1	–	–

* = No MIC values were provided by the machine, − No resistance and/or intermediate isolate

## Discussion

Rapid and accurate identification of *Candida* species down to the species level is of great importance for the selection of appropriate antifungal agents and for patient management. In the present study, 18 different species of yeasts compromising of 15 *Candida* species and three yeasts other than *Candida* species were identified. While *Candida albicans* was the most frequently isolated yeast in the present study, its prevalence was lower than a study conducted in Northwest Ethiopia [16] and higher than a study conducted in central Ethiopia [17]. *Candida albicans* as the most frequently isolated species was also reported by Sasso et al [30], Mnge et al [31], and Zeng et al [32]. *C. parapsilosis* as the most frequently isolated yeast was depicted in a study conducted by Sahal and Bilkay [33].

A preponderance of *C. albicans* compared with NAC species varies between and with countries. A preponderance of *C. albicans* was observed in our study. Our finding was consistent with previous studies conducted elsewhere [16–18, 21–23, 25–27]. However, a preponderance of NAC species were demonstrated by many earlier studies [19, 20, 24, 28, 29]. While *C. albicans* is still the predominant species in Ethiopian studies, the occurrence of NAC species in the current study was higher than those studies [16, 17] underlining the shifting trend of *Candida* infections towards NAC species. A shift of *Candida albicans* towards non-*albicans Candida* species observed in the present study could be due to an improved detection rate of non-*albicans Candida* species or a true prevalence change. Our result is in line with a study conducted by Ghazi et al [34] that demonstrated a shift of *Candida albicans* towards non-*albicans Candida* species in the Middle East and North Africa. In the present study, *C. krusei* was the 2nd dominant species. Our result was in line with that of Bitew and Abebaw [17] and Mohndas and Ballal [19], but in contradiction with many other studies where *C. glabrata* or *C. tropicalis* was reported as a 2nd predominant NAC species [16, 18–20, 22, 23, 25–29]. A choice of fluconazole as a first line drug for the management of candidiasis in Ethiopia [35] may be a risk factor for a higher isolation rate of *C. krusei* in this study. Treatment of fungal infections by azole antifungals such as fluconazole was suggested to enhance the selection of resistant *Candida* species by shifting infection and/or colonization to more intrinsically resistant species chiefly of *C. krusei* or *C. glabrata* [36]*.* The lower isolation rate of *C. glabrata* compared to *C. krusei* which is also intrinsically resistant to fluconazole in the current study is unclear. The geographical difference can be a contributing factor for differences in the distribution of NAC species is suggested by Falags et al [37]. This can be demonstrated by the fact that the prevalence of *C. glabrata* is high in America, North and Central Europe while *C. tropicalis* is commonly isolated in South America and Asia [37]. *C. famata* was the third predominant *Candida* species in the present study. Similar results were obtained by many previous studies [38–40]. These studies have demonstrated that strains of *C. guilliermondii*, *C. lusitaniae*, *C. fermentati*, *C. intermedia*, and *C. palmioleophila* have been reported as *C. famata* by phenotypic methods such as the VITEK 2 compact system, and such drawback of the VITEK 2 could be a possible explanation for the higher isolation rate of *C. famata* in the current study. Therefore, *Candida* species identified as *C. famata* by phenotypic methods should be confirmed by molecular methods.

In the present study, the drug sensitivity patterns of all yeast isolates were tested against five antifungal drugs. The MIC for *C. famata* to all the drugs tested and *C. ciferrii* for echinocandins were not provided by the VITEK. This could probably be due to the fact that the MIC values of the drugs against the isolates are not present in the system database and/or the VITEK 2 system may take a longer incubation period to determine MIC endpoints of the drugs against the isolates.

In our study, 85.6, 10.5, and 3.9% of *Candida* species were susceptibly, resistant and, intermediated to fluconazole, respectively. In view of the overall high level of susceptibility of *Candida* species to the antifungal demonstrated that the drug is still considered to be an active drug against *Candida* species, regardless of the indiscriminate use of the agent for therapy and prevention in Ethiopia [35]. However, *C. krusei* recognized to be a naturally non-susceptible to fluconazole [41] was found out to be 100% resistant to it. Our result was compatible with our previous study [17] and another study [42] but in disagreement with the result of Elfeky et al [18] who reported that as high as 60% of the species are susceptible to the agent. Based on our in vitro drug susceptibility testing result, we suggest a search for alternative agents is essential when treating fungal infections caused by *C. krusei*. Voriconazole was the most active drug against yeast isolates among all drugs tested. All yeast isolates were 100% susceptible to the antifungal except one species of *C. ciferrii* which was resistant to the agent giving the overall percentage susceptibility of 99.4%. While inhibition of ergosterol synthesis is a common mode of action to all azole antifungal drugs [42) susceptibility difference between fluconazole and voriconazole against *C. krusei in* our study is unclear*.* On the other hand, contrary to the notion that *C. glabrata* is intrinsically non-susceptible to fluconazole, all isolates in our study were sensitive to all azole drugs tested. More or less similar results were reported by many studies [17, 27, 43].

In our previous study [17], the in vitro susceptibility of all yeast isolates were 100% to both micafungin and caspofungin. However, in this study a resistance rate of 4% to each drug was noted. *C. rugosa* (20%), *C. lipolytica* (16.7%), and *C. albicans* (3.8%) were the isolates where resistance strains to the two drugs were observed. While the number of isolates categorized as susceptible, intermediate, and resistance to both drugs were the same, micafungin was more potent than caspofunginn supporting the work of Ostrosky-Zeichne et al [44]. Further, while the mode of action of the two antifungals is the same that is preventing fungal cell wall synthesis by inhibiting the enzyme that synthesizes β-glucan [36], a discrepancy in potency observed in our study is not clear.

Of 181 *Candida* isolates in which MIC values provided by the machine 86.2, 6.6% and, 7.1% were susceptible, resistant, and intermediate to flucytosine, respectively. Surprisingly, about 78.6% of *C. krusei* was resistant to the drug. Percentage drug resistance noted in this study was more than two fold than from that of our previous study [17]. Regarding the drug resistance profile of yeast other than Candida species no MIC values were obtained for both *C. laurentii* and *T. mucoid. C. neoformans* was 100% susceptible to fluconazole and flycytosine, but MIC values were not obtained against the remaining drugs tested.

Although attempts were made to collect demographic data and history of each patient from request forms filled out by physicians, unfortunately, the recording of these parameters in the test request form was not consistent. Consequently, demographic data and the clinical history of each patient were not documented for this study. The mechanisms responsible for the acquisition of antifungal resistance in our clinical isolates were not determined due to resource shortage and this can be considerd as another limitation of this study.

## Conclusions

The present study demonstrated the distribution of *Candida* species in different clinical specimens where the isolation rate of non-*albicans Candida* species was comparable to *Candida albicans*. The high resistance rate of *C. krusei* to fluconazole and flucytosine may demonstrate that the treatment of candidiasis empirically is questionable.

## Data Availability

The datasets during and/or analyzed during the current study are available from the corresponding author and can be obtained up on reasonable request.

## Abbreviation

AES: Advanced expert system
ATCC: American Type Culture collection
DRERC: Department Research Ethical Review Committee
IRB: Internal Review Board
MIC: Minimum Inhibitory Concentration
NAC: Non- albican candidia
UK: United Kingdom

## References

[1] Zaoutis TE, Argon J, Chu J, Berlin JA, Walsh TJ, Feudtner C (2005). The epidemiology and attributable outcomes of candidemia in adults and children hospitalized in the United States: a propensity analysis. Clin Infect Dis.

[2] Colombo AL, Nucci M, Park BJ, Nouér SA, Arthington-Skaggs B, da Matta DA (2006). Epidemiology of candidemia in Brazil: a nationwide sentinel surveillance of candidemia in eleven medical centers. J Clin Microbiol.

[3] Wilcox CM, Straub RF, Clark WS (1995). Prospective evaluation of oropharyngeal findings in human immunodeficiency virus-infected patients with esophageal ulceration. American J gastroenterol.

[4] Snydman DR (2003). Shifting in patterns in the epidemiology of nosocomial Candida infections. Chest.

[5] Sobel JD (2006). The emergence of non-albicans Candida species as causes of invasive candidiasis and candidemia. Curr Infct Dis Rep.

[6] Latha R, Sasikala R, Muruganandam RN, Babu RY (2011). Study on the shifting patterns of Non Candida albicans Candida in lower respiratory tract infections and evaluation of the CHROMagar in identification of the Candida species. J Microbiol Biotech Res.

[7] Knoke M, Schulz K, Bernhardt H (1997). Dynamics of *Candida* isolations from humans from 1992-1995 in Greifswald. Germany Mycoses.

[8] Shin JH, Lim WH, Shin DH, Suh SP, Ryang DW (1999). *Candida* species isolated from clinical specimens and medical personnel. Korean J Infect Dis.

[9] Upton A, Marr AK (2006). Emergence of opportunistic mold infections in the hematopoietic stem cell transplant patient. Curr Infect Dis Rep.

[10] Marchetti O, Bille J, Fluckiger U, Eggimann P, Ruef C, Garbino J (2004). Epidemiology of candidemia in Swiss tertiary care hospitals: secular trends, 1991–2000. Clin Infect Dis.

[11] Yapar N (2014). Epidemiology and risk factors for invasive candidiasis. Ther Clin Risk Manag.

[12] Sievert DM, Ricks P, Edwards JR, Schneider A, Patel J, Srinivasan A (2013). Antimicrobial-resistant pathogens associated with healthcare-associated infections: summary of data reported to the national healthcare safety network at the centers for disease control and prevention, 2009–2010. Infect Control Hosp Epidemiol.

[13] Pfaller M. A., Diekema D. J., Messer S. A., Boyken L., Hollis R. J. (2003). Activities of Fluconazole and Voriconazole against 1,586 Recent Clinical Isolates of Candida Species Determined by Broth Microdilution, Disk Diffusion, and Etest Methods: Report from The ARTEMIS Global Antifungal Susceptibility Program, 2001. Journal of Clinical Microbiology.

[14] Zaragoza O, Mesa-Arango AC, Gomez-Lopez A, Bernal-Martinez L, Rodriguez-Tudela JL (2011). A Process Analysis of Variables for Standardization of Antifungal Susceptibility Testing of Non-Fermentative Yeasts. Antimicrob Agents Chemother.

[15] Pfaller MA, Diekema DJ, Procop GW, Rinaldi MG (2007). Multicenter comparison of the VITEK 2 yeast susceptibility test with the CLSI broth microdilution reference method for testing fluconazole against *Candida* spp. J Clin Microbiol.

[16] Mulu A, Kassu A, Anagaw B, Moges B, Gelaw A, Alemayehu M (2013). Frequent detection of ‘azole’ resistant Candida species among late presenting AIDS patients in northwest Ethiopia. BMC Infect Dis.

[17] Bitew A, Abebaw Y (2018). Vulvovaginal candidiasis: Species distribution of *Candida* and their antifungal susceptibility pattern. BMC Womens Health.

[18] ElFeky DS, Gohar NM, El-Seidi EH, Ezzat MM, Hassan S, AboElew SH (2016). Species identification and antifungal susceptibility pattern of Candida isolates in cases of vulvovaginal candidiasis. Alexandria J Med.

[19] Mohandas V, Ballal M (2011). Distribution of *Candida* Species in different clinical samples and their virulence: Biofilm formation, proteinase and phospholipase production: A study on hospitalized patients in Southern India. J Glob Infec Dis.

[20] Das KH, Getso MI, Azeez-Akande O (2016). Distribution of *Candida albicans* and non-albicans Candida *in clinical* samples and their intrinsic biofilm production status. Int J Med Sci Public Health.

[21] Sida H, Pethani J, Dalal P, Hiral SH (2017). Study of Changing Trend in the Clinical Distribution of Candida Species in Various Clinical Samples at Tertiary Care Hospital, Ahmedabad, Gujarat. Ntl J Community Med.

[22] Muadcheingka T, Tantivitayakul P (2015). Distribution of *Candida albicans* and non-albicans Candida species in oral candidiasis patients: Correlation between cell surface hydrophobicity and biofilm forming activities. Archive oral biol.

[23] Chi H-W, Yang Y-S, Shang S-T, Chen K-H, Yeh K-M, Chang F-Y (2011). *Candida albicans* versus non-albicans bloodstream infections: The comparison of risk factors and outcome. J Microbiol, Immun Infec.

[24] Kaur R, Dhakad MS, Goyal Kumar RR (2016). Emergence of non-albicans Candida species and antifungal resistance in intensive care unit patients. Asian Pac J Trop Biomed.

[25] Ribeiro ALR, de Melo A-JS, de Menezes SF, Silvia Helena Marques-da-Silva SH, ACR RV, de Alencar Menezes TO (2015). Oral carriage of Candida species in HIV-infected patients during highly active antiretroviral therapy (HAART) in Belém, Brazil. Oral Surg Oral Med Oral Pathol Oral Radiol.

[26] Schmalrec AF, Willinger B, Haase BG, Lass-Flo C, Feeler K (2012). Species and susceptibility distribution of 1062 clinical yeast isolates to azoles, echinocandins, flucytosine and amphotericin B from a multi-centre study. Mycoses.

[27] Richter S. S., Galask R. P., Messer S. A., Hollis R. J., Diekema D. J., Pfaller M. A. (2005). Antifungal Susceptibilities of Candida Species Causing Vulvovaginitis and Epidemiology of Recurrent Cases. Journal of Clinical Microbiology.

[28] Badiee P, Alborzi A (2011). Susceptibility of clinical *Candida* species isolates to antifungal agents by E-test, Southern Iran: A five year study. Iran J MicrobioL.

[29] Godoy P, Tiraboschi IN, Severo LC, Beatriz Bustamante B, Calvo B, de Almeida LP (2003). Species Distribution and Antifungal Susceptibility Profile of *Candida* spp. Bloodstream Isolates from Latin American Hospitals**.***Mem Inst Oswaldo Cruz*. Rio de Janeiro.

[30] Sasso M, Roger C, Sasso M, Poujol H, Saber Barbar S, Lefrant J (2017). Changes in the distribution of colonising and infecting *Candida* spp. isolates, antifungal drug consumption and susceptibility in a French intensive care unit: A 10-year study. Mycoses.

[31] Mnge P, OkeleyeBI, Vasaikar SD, Apalata T. Species distribution and antifungal susceptibility patterns of Candida isolates from a public tertiary teaching hospital in the Eastern Cape Province, South Africa. Braz J Med Biol Res. 2016. 10.1590/1414-431X20175797. 10.1590/1414-431X20175797 PMC5479383 28513771

[32] Zeng Z, Tian G, Ding Y, Yang K, Liu J, Deng J (2019). Surveillance study of the prevalence, species distribution, antifungal susceptibility, risk factors and mortality of invasive candidiasis in a tertiary teaching hospital in Southwest China. BMC Infect Dis.

[33] Sahal G, Bilkay IS (2018). Distribution of clinical isolates of *Candida* spp. and antifungal susceptibility of high biofilm-forming *Candida* isolates. Rev Soc Bras Med Trop.

[34] Ghazi S, Rafei R, Osman M, Safadi EI, Mallat H, Papon N (2019). The epidemiology of Candida species in the Middle East and North Africa. J Mycol Med.

[35] Guidelines for management of opportunistic infections and antiretroviral treatment in adolescents and adults in Ethiopia. Addis Ababa Ethiopia: Federal HIV/AIDS Prevention and Control Office Federal Ministry of Health; 2007.

[36] Alexander BD, Perfect JR (1997). Antifungal resistance trends towards the year 2000: implications for therapy and new approaches. Drugs.

[37] Falagas ME, Roussos N, Vardakas KZ (2010). Relative frequency of albicans and the various non-albicans Candida spp. among candidemia isolates from inpatients in various parts of the world: a systematic review. Int J Infect Dis.

[38] Desnos-Ollivier M, Ragon M, Robert V, Raoux D, Gantier JC, Dromer F (2008). Debaryomyces hansenii (Candida famata), a rare human fungal pathogen often misidentified as *Pichia guilliermondi*i (*Candida guilliermondii*). J Clin Microbiol.

[39] Jensen RH, Arendrup MC (2011). *Candida palmioleophila*: characterization of a previously overlooked pathogen and its unique susceptibility profile in comparison with five related species. J Clin Microbiol.

[40] Mariana Castanheira A, Leah N, Woosley A, Daniel J, Diekema B, Ronald N (2013). *Candida guilliermondii* and Other Species of *Candida* Misidentified as *Candida famata*: Assessment by Vitek 2, DNA Sequencing Analysis, and Matrix-Assisted Laser Desorption Ionization–Time of Flight Mass Spectrometry in Two Global Antifungal Surveillance Programs. J Clin Microbiol.

[41] Lyon G. M., Karatela S., Sunay S., Adiri Y. (2010). Antifungal Susceptibility Testing of Candida Isolates from the Candida Surveillance Study. Journal of Clinical Microbiology.

[42] Rex J, Stevens D, Mandell G, Bennett J, Dolin R (2005). Systemic antifungal agents. Principles and practice of infectious diseases, vol. 1.

[43] Hasanvand S, Qomi HA, Kord M, Didehdar M (2017). Molecular epidemiology and in vitro antifungal susceptibility of *Candida* isolates from women with vulvovaginal candidiasis in northern cities of Khuzestan Province. Iran Jundishapur J Microbiol.

[44] Ostrosky-Zeichne LA, Lashof MO, Kullberg BJ, Rex JH (2003). Voriconazole salvage treatment of invasive candidiasis. Eur J Clin Microbiol Infect Dis.

